# Human Microbiome as an Immunoregulatory Axis: Mechanisms, Dysbiosis, and Therapeutic Modulation

**DOI:** 10.3390/microorganisms13092147

**Published:** 2025-09-14

**Authors:** Matías Cortés, Paula Olate, Rodrigo Rodriguez, Rommy Diaz, Ailín Martínez, Genisley Hernández, Nestor Sepulveda, Erwin A. Paz, John Quiñones

**Affiliations:** 1 Carrera de Biotecnología, Universidad de La Frontera, Temuco 4811230, Chile; m.cortes10@ufromail.cl (M.C.); p.olate02@ufromail.cl (P.O.); 2 Centro de Tecnología e Innovación de La Carne, Universidad de la Frontera, Temuco 4780000, Chile; rommy.diaz@ufrontera.cl (R.D.); a.martinez26@ufromail.cl (A.M.); nestor.sepulveda@ufrontera.cl (N.S.); 3 Biocontrol Research Laboratory, Universidad de La Frontera, Temuco 4811230, Chile; r.rodriguez09@ufromail.cl; 4 Facultad de Ciencias Agropecuarias y Medioambiente, Universidad de La Frontera, Temuco 4780000, Chile; genisleyh@gmail.com; 5 Doctoral Program in Science Major in Applied Cellular and Molecular Biology, Universidad de La Frontera, Av. Francisco Salazar 01145, Temuco 4811230, Chile; 6 UWA Institute of Agriculture, The University of Western Australia, Perth 6009, Australia; erwin.pazmunoz@uwa.edu.au

**Keywords:** human microbiome, immunomodulation, dysbiosis, microbiome-based therapies, immune homeostasis

## Abstract

The human microbiome plays a central role in modulating the immune system and maintaining immunophysiological homeostasis, contributing to the prevention of immune-mediated diseases. In particular, the gut microbiota is a key ecosystem for immune system maturation, especially in early life. This review aimed to analyze the molecular and cellular mechanisms linking the microbiome to immune and neuronal functions, as well as the impact of dysbiosis and emerging therapeutic strategies targeting the microbiome. The analysis was based on scientific databases, prioritizing studies published since 2000, with special emphasis on the past decade. The microbiome influences immune signaling through microorganism-associated molecular patterns (MAMPs) and pattern recognition receptors (PRRs), including Toll-like receptors (TLRs). Additionally, microbial metabolites—such as short-chain fatty acids (SCFAs), tryptophan derivatives, and secondary bile acids—exert significant immunomodulatory effects. The intestinal epithelial barrier is also described as an active immunological interface contributing to systemic regulation. The literature highlights innovative therapies, including fecal microbiota transplantation (FMT), probiotics, and microbiome editing with CRISPR-Cas technologies. These strategies aim to restore microbial balance and improve immune outcomes. The growing body of evidence positions the microbiome as a valuable clinical and diagnostic target, with significant potential for application in personalized medicine.

## Simple Summary

The microorganisms that live in our body, especially those in the gut, are essential for keeping the immune system balanced and protecting against disease. During the first years of life, these microbes help the immune system to grow and work properly. This review explored how the microbiome communicates with the immune system and the brain, what happens when this balance is disturbed, and which new treatments may help restore it. We found that gut microbes send signals to the immune system through different molecules and produce substances that can reduce inflammation and support health. The intestinal lining also plays an important role as a barrier that protects the body and helps regulate immunity. Promising therapies are being developed to repair imbalances in the microbiome, including the use of beneficial bacteria, stool transplants, and advanced genetic tools. These strategies aim to improve health by restoring a healthy microbial community. Understanding and using this knowledge could help create more personalized treatments for immune-related diseases, offering safer and more effective options for patients while improving overall well-being in society.

## 1. Introduction

The human body harbors a diverse and complex community of microorganisms known as the microbiome, composed primarily of bacteria but also including viruses, fungi, and archaea. This microbial network colonizes multiple anatomical niches, including the gastrointestinal tract, skin, respiratory tract, urogenital system, and oral cavity, establishing unique ecosystems adapted to each environment [1]. The composition and abundance of the microbiome vary significantly among individuals and across body sites, influenced by genetic factors, diet, lifestyle, age, and antibiotic use.

Among these ecosystems, the gut microbiome has been the most extensively studied due to its high microbial density and its central role in immunological and metabolic processes. Other microbiomes, such as those of the skin, oral cavity, respiratory tract, and urogenital system, also contribute to immune surveillance, immunological tolerance, and infection prevention [2]. Beyond its role in digestion, the microbiome actively participates in the development and maturation of the immune system, particularly during early life. Early exposure to a diverse microbiota is essential for educating the immune system, promoting tolerance toward commensal microorganisms, and aiding in the distinction between self and non-self.

The integrity of the intestinal epithelial barrier, partially modulated by the microbiota, is crucial for preventing bacterial translocation and inappropriate immune activation. Additionally, microbial metabolites such as short-chain fatty acids (SCFAs; including butyrate, propionate, and acetate) exert significant immunomodulatory effects, influencing the differentiation and function of various immune cells [3]. This bidirectional interaction between the microbiome and the immune system is both dynamic and complex: while the immune system shapes microbial composition through selective pressures, the microbiome, in turn, affects the development and functionality of immune cells at both local and systemic levels.

An imbalance in the composition and function of the microbiome, known as dysbiosis, has been associated with a wide range of diseases. These include inflammatory bowel diseases such as Crohn’s disease and ulcerative colitis [4], allergies, autoimmune disorders, inflammation-driven cancers, and neurological conditions. Recent studies have identified specific microbial patterns in diseases such as systemic lupus erythematosus [5], rheumatoid arthritis [6], systemic sclerosis [7], antiphospholipid syndrome [8], and tuberculosis [9]. Furthermore, dysbiosis has been investigated in the context of chronic inflammation in people living with HIV [10] and its potential role in predisposing individuals to type 2 diabetes.

Of interest is the connection between the microbiome and the central nervous system via the gut–brain axis, which involves neural, immune, and endocrine pathways. The gut microbiota can influence brain function through the production of neurotransmitters and the modulation of systemic inflammation, opening new research avenues in neurological and psychiatric disorders [11]. In-depth characterization of the microbiome and its interaction with the immune system offers valuable clinical opportunities. The identification of microbial profiles as diagnostic or prognostic biomarkers, along with targeted microbiome modulation through probiotics, prebiotics, or fecal microbiota transplantation, represents a promising therapeutic approach. In this context, microbiome research not only deepens our understanding of human physiology but also paves the way for innovative preventive and therapeutic strategies for numerous diseases.

Therefore, this review aimed to analyze the molecular and cellular mechanisms linking the microbiome to immune function, as well as the effects of dysbiosis and recent therapeutic strategies aimed at its modulation.

## 2. Materials and Methods

### 2.1. Selection Criteria

This review was conducted using a systematic and structured approach aimed at ensuring transparency, reproducibility, and methodological rigor. To this end, an exhaustive search was conducted in high-impact biomedical and multidisciplinary databases (PubMed/MEDLINE, Scopus, Web of Science, Embase, ScienceDirect, SpringerLink, Wiley Online Library, and DOAJ) between January 2020 and April 2025, supplemented by specialized repositories in microbiology and immunology, also considering some highly cited previous articles of conceptual relevance. The search strategy was based on MeSH descriptors and combinations of keywords using Boolean operators (e.g., “gut microbiome AND immunity,” “dysbiosis AND inflammation AND chronic diseases,” “microbiome AND autoimmune diseases,” “gut-brain axis AND immunity,” among others). The inclusion criteria covered research articles (clinical, observational, and experimental in humans and animal models), systematic reviews, meta-analyses, and narrative reviews published in Q1–Q2 indexed journals, available in English or Spanish, and explicitly addressing the microbiome-immunity interaction, the effects of dysbiosis, or microbial modulation strategies. Publications not subject to peer review, studies with insufficient samples, duplicate articles, works focused exclusively on microbial taxonomy without an immunological relationship, and editorial-type documents or technical notes without complete data were excluded. The selection process involved identifying 3264 initial records, screening titles and abstracts to leave 587 preselected articles, and reviewing the full text to result in 180 included studies. A standardized template was used to extract information, collecting variables on design, population, microbial alterations, metabolites involved, immunological mechanisms, and main findings. The synthesis of evidence was structured around five thematic areas: microbial signaling and immune recognition; microbial metabolites and immunoregulation; epithelial barrier integrity and immune homeostasis; dysbiosis and its relationship with inflammatory, autoimmune, metabolic, neurological, and infectious diseases; and finally, therapeutic and translational perspectives, including probiotics, prebiotics, postbiotics, fecal transplantation, immunotherapy, and personalized medicine. The organization of the content combined a progressive narrative, comparative tables, and conceptual figures illustrating key mechanisms, while the critical analysis prioritized methodological quality, consistency, and level of evidence of the studies, highlighting convergences, discrepancies, and possible hypotheses for future research.

### 2.2. Composition and Function of the Human Microbiome

The human microbiome is defined as a dynamic community of microorganisms that colonizes various surfaces and cavities of the human body. This complex biological network is composed of bacteria, archaea, fungi, viruses, and certain eukaryotes, which interact symbiotically with the host, establishing relationships that range from mutualism and commensalism to, in some contexts, opportunistic pathogenicity [2]. Far from being a passive microbial collection, the microbiome plays essential roles in multiple physiological processes of the host organism [12].

Current scientific evidence acknowledges its active participation in the maturation and regulation of the immune system, protection against pathogens, maintenance of immune tolerance, and modulation of inflammatory responses [13]. Moreover, the microbiome directly influences key metabolic pathways by facilitating the digestion of complex compounds, synthesizing vitamins, producing bioactive metabolites, and extracting energy from substrates indigestible by the host [14].

Microbial composition and diversity vary substantially among individuals and are shaped by genetic, dietary, environmental, and behavioral factors [15]. Additionally, within a single organism, each ecological niche, such as the gut, skin, vagina, or oral cavity, presents unique microbial profiles determined by specific local conditions, including pH, temperature, humidity, nutrient availability, and host secretions.

Maintaining a dynamic balance between these microbial communities and their physiological environment is essential for preserving the overall homeostasis of the organism. Disruptions to this equilibrium, known as dysbiosis, have been associated with a wide range of diseases, including gastrointestinal disorders, autoimmune diseases, metabolic syndromes, and neuropsychiatric conditions [16]. Ongoing research continues to unravel the depth of host–microbiome interactions, positioning this community as a strategic target for the development of new diagnostic tools, preventive strategies, and personalized therapies [17].

### 2.3. Main Microbial Niches in the Human Body

#### 2.3.1. Gut Microbiome

The human gut harbors the densest and most diverse microbial community in the body, forming a complex ecosystem composed of trillions of microorganisms from thousands of species. Among the most representative bacterial phyla are *Firmicutes* and *Bacteroidetes*, whose proportions vary depending on factors such as habitual diet, host genetics, and physiological status [14]. Within Firmicutes, genera such as *Faecalibacterium* (a butyrate producer with anti-inflammatory effects), *Clostridium* (involved in complex carbohydrate fermentation), and *Lactobacillus* (known for lactic acid production and local immune modulation) are prominent. On the other hand, Bacteroides, a dominant genus among *Bacteroidetes*, plays a key role in the degradation of non-digestible polysaccharides, releasing nutrients that benefit both the microbiome and the host [18].

The functional composition of the gut microbiome is highly plastic and responsive to various influences, with diet being one of the most critical modulatory factors. Changes in fiber intake, macronutrient distribution, or consumption of bioactive compounds can alter the abundance of microbial groups and their associated metabolic pathways [18]. Similarly, antibiotic use can induce persistent dysbiosis, reducing microbial diversity and stability [19]. Current research is exploring how these alterations contribute to diseases such as inflammatory bowel disease, obesity, type 2 diabetes, and irritable bowel syndrome, as well as potential interventions using probiotics, prebiotics, dietary modifications, or fecal microbiome transplantation [18].

#### 2.3.2. Skin Microbiome

The skin, as the body’s primary physical barrier to the external environment, hosts microbial communities adapted to diverse microenvironments defined by local factors such as moisture, pH, sebum secretion, temperature, and cellular desquamation. Dominant phyla include Actinobacteria, Firmicutes, Proteobacteria, and Bacteroidetes [20]. In dry regions such as the forearms, Actinobacteria predominate, including *Corynebacterium*, which is involved in the production of skin-protective enzymes. In moist areas, such as the axillae and skin folds, *Staphylococcus* species (*Firmicutes*) are more common, while *Pseudomonas* (*Proteobacteria*) is present in various regions and may act as a commensal or opportunistic pathogen [21].

The skin microbiome not only provides physical protection but also plays a critical immunological role: it competes with pathogens for nutrients and adhesion sites, produces antimicrobial substances, and modulates both innate and adaptive immune responses [22]. Disruptions in this balance have been associated with dermatological conditions such as atopic dermatitis, acne, and psoriasis, highlighting the microbiome’s importance as a key component of skin health [21].

#### 2.3.3. Vaginal Microbiome

Unlike other microbial niches, the vaginal microbiome is characterized by relatively low microbial diversity but highly specialized and dynamic functions. In healthy women of reproductive age, species from the *Lactobacillus* genus, particularly *L. crispatus*, *L. gasseri*, *L. iners*, and *L. jensenii*, predominate. These bacteria ferment epithelial glycogen to produce lactic acid, maintaining an acidic vaginal pH (3.5–4.5) that inhibits the growth of pathogenic microorganisms [17]. Some species also produce bacteriocins with targeted antimicrobial activity [23]. The stability of the vaginal microbiome is influenced by hormonal fluctuations, sexual activity, hygiene practices, and antibiotic use. Vaginal dysbiosis, marked by a reduction in *Lactobacillus* and an increase in anaerobic bacteria such as *Gardnerella vaginalis*, has been associated with bacterial vaginosis, sexually transmitted infections, and obstetric complications [24]. Current research seeks to understand the determinants of dysbiosis and develop strategies for restoring vaginal microbial balance.

#### 2.3.4. Oral Microbiome

The oral cavity constitutes one of the most complex ecosystems in the human body, comprising multiple microhabitats, including the tongue, gums, palate, and dental surfaces, that host a rich and diverse microbial community [25]. Over 700 bacterial species have been identified in this niche, spanning phyla such as *Firmicutes*, *Actinobacteria*, *Bacteroidetes*, *Proteobacteria*, and *Fusobacteria* [15]. Prominent genera include *Streptococcus*, which plays a key role in the initial formation of biofilms; *Actinomyces*, which adheres to tooth surfaces; *Prevotella*, commonly found in subgingival biofilms; and *Veillonella*, known for metabolizing lactate produced by other bacteria.

A central feature of the oral microbiome is its ability to form structured biofilms, such as dental plaque, where microorganisms coexist in a complex extracellular matrix, engaging in both cooperative and competitive interactions [26]. Dysbiosis within these communities, often driven by high-sugar diets or poor oral hygiene, can promote the development of dental caries and periodontal diseases [27]. Moreover, an increasing body of evidence links the oral microbiome to systemic conditions, including cardiovascular diseases, diabetes, and pregnancy-related complications [28].

#### 2.3.5. Saliva

Saliva plays a crucial role in shaping the oral ecosystem by providing nutrients, antimicrobial enzymes such as lysozyme and lactoperoxidase, and supporting the clearance of non-adherent microorganisms [25]. The lactoperoxidase system generates hypothiocyanite (OSCN^−^), a potent antimicrobial that reduces plaque formation and gingival inflammation, while lysozyme contributes by lysing gram-positive bacteria, limiting their pathogenicity [26]. Moreover, salivary proteins, including mucins and peroxidases secreted by the submandibular and sublingual glands, are essential for maintaining microbial diversity, and alterations in these components have been linked to dysbiosis and systemic diseases [27]. Altogether, saliva not only nourishes and protects but also regulates the oral microbiome, consolidating its role as a biomarker and therapeutic target in oral and systemic health.

#### 2.3.6. Role of the Microbiome in Metabolism, Protective Effects, and Trophic Function

The human microbiome performs essential metabolic functions that complement the physiological capabilities of the host. In particular, the gut microbiome stands out for its ability to degrade compounds that escape digestion in the small intestine, such as complex polysaccharides and dietary fiber. Through anaerobic fermentation, these bacteria produce short-chain fatty acids (SCFAs; including butyrate, propionate, and acetate), which serve multiple important functions [29]. Beyond providing an energy source for colonocytes, SCFAs are involved in the regulation of inflammation, glycemic homeostasis, and lipid metabolism, interacting with specific host receptors and modulating systemic signaling pathways [30].

The gut microbiome contributes to the biosynthesis of essential micronutrients. Among the most notable are vitamin K (critical for coagulation) and several B-complex vitamins, which play enzymatic roles in energy metabolism and neurological function [31]. This biosynthetic capacity enhances the nutritional repertoire of the human host, especially under conditions of dietary limitation.

From an immunological perspective, the microbiome provides protective effects against colonization by pathogenic microorganisms. It achieves this through mechanisms such as competition for nutrients and ecological niches, production of antimicrobial compounds (e.g., bacteriocins), and stimulation of local immune responses that strengthen mucosal defenses [22]. The integrity of the intestinal barrier, modulated by a healthy microbiota, is another cornerstone in preventing the translocation of pathogens or their toxic products into systemic circulation [32]. The induction of protective mucus reinforces this barrier effect, the expression of tight junction proteins between enterocytes, and the modulation of epithelial function [33].

Due to its metabolic and defensive functions, the microbiome plays a crucial trophic role in the development and maintenance of the gastrointestinal tract. Fermentation products not only nourish epithelial cells but also promote their proliferation, differentiation, and repair, thereby preserving intestinal mucosal integrity [14]. At the immunological level, the continuous exposure of the intestinal immune system to microbial antigens fosters the maturation of immunological tolerance mechanisms. This process allows discrimination between commensal microorganisms (which should be tolerated) and pathogens (which require an appropriate immune response) [34].

Structural components of the microbiome, such as lipopolysaccharides (LPS) and peptidoglycans, are recognized by innate immune receptors (such as Toll-like receptors), triggering signaling cascades that lead to the production of cytokines and immunological mediators. This constant interaction between the microbiome and local immunity is essential for maintaining a functional equilibrium that supports host protection while preventing inappropriate immune responses [2].

#### 2.3.7. Development of the Immune System and Its Interaction with the Microbiome

The human immune system does not emerge spontaneously or in isolation; rather, its development is a dynamic, sequential, and tightly regulated process. It begins during intrauterine life and continues to mature throughout infancy, adolescence, and even into adulthood. A fundamental player in this process is the microbiome, a complex and diverse ecosystem of microorganisms that colonizes the human body, particularly the gastrointestinal tract, from the earliest stages of life [35]. The relationship between the developing immune system and the microbiome is not merely coexistent; it is a reciprocal and continuous interaction that is essential for the establishment of immune homeostasis. This homeostasis enables the organism to distinguish between self and non-self, mount effective responses against pathogens, and simultaneously maintain immunological tolerance toward commensal microorganisms and self-antigens [36].

During the early years of life, this symbiotic interaction shapes the architecture and function of both primary and secondary lymphoid organs, influences the differentiation and activation of multiple immune cell populations, and lays the foundation for robust, adaptable, and long-lasting immunity [37]. At this critical stage, signals from the microbiome guide the education of the immune system, teaching it to respond with balance and efficiency [38].

Recent scientific evidence has begun to unravel the complexity of this connection, revealing that imbalances in the composition or function of the early microbiome can have lasting repercussions on immune health, increasing susceptibility to inflammatory, allergic, autoimmune, and even metabolic diseases [39].

A deeper understanding of this molecular dialogue between host and microbiome not only enriches our view of immune development but also opens the door to innovative interventions, such as targeted probiotics, diet-based modulation, and birth or breastfeeding strategies aimed at promoting optimal immune health from the very first days of life.

### 2.4. Microbial Colonization at Birth

Microbial colonization of the new-born marks a fundamental biological event that initiates a complex symbiotic relationship between the human host and microorganisms, with long-lasting implications for immune development and lifelong health [40]. This process begins immediately at birth, when the neonate first encounters external microbial reservoirs [41].

In vaginal deliveries, the newborn is exposed to the maternal birth canal microbiome, predominantly comprising *Lactobacillus*, *Prevotella*, and *Sneathia*, which initiate colonization of the infant’s gastrointestinal tract [42]. In contrast, infants born by caesarean section are initially colonized by microorganisms from maternal skin and the hospital environment, resulting in a distinct and less diverse microbial profile during early life. These early differences, although subtle, can influence the establishment of the intestinal microbiome and may have long-term consequences on immune programming [43].

Neonatal microbiome formation is not a static event but rather a dynamic, ongoing process influenced by several postnatal factors, including mode of delivery, feeding type (exclusive breastfeeding vs. formula), early antibiotic exposure, hygiene conditions, and household environment [44]. Among these, breast milk stands out not only as a nutritional source but also as a key immunomodulatory element [45]. It contains maternal immunoglobulins (primarily secretory IgA), specific oligosaccharides such as HMOs, cytokines, and growth factors that contribute to the maturation of the infant’s immune system and promote the growth of beneficial bacteria such as *Bifidobacterium* and *Lactobacillus* [39].

This initial colonization process establishes a continuous and bidirectional dialogue with the immature immune system. Microbiome-derived molecular signals guide immune cell differentiation and activation, foster immunological tolerance, and prepare the organism for future responses to external agents [35]. Ongoing research continues to reveal the sensitivity of this critical period and how early disruptions in microbial colonization (whether clinical or environmental) can shape divergent immune trajectories, potentially increasing susceptibility to chronic, allergic, or autoimmune diseases later in life.

### 2.5. Maturation of the Innate and Adaptive Immune System

The maturation of the human immune system is profoundly influenced by constant and multifaceted interactions with the microbiome, a symbiotic relationship essential for both innate and adaptive immunity [36]. From the earliest stages of life, conserved microbial components, known as microbe-associated molecular patterns (MAMPs), are recognized by pattern recognition receptors (PRRs) on innate immune cells, including macrophages, dendritic cells, and natural killer (NK) cells [46].

These interactions activate complex intracellular signaling networks that trigger the production of both pro- and anti-inflammatory cytokines, chemokines, and other effector molecules. In turn, these mediators finally modulate the inflammatory response at both local and systemic levels, ensuring a rapid and effective defense against pathogens while avoiding excessive or chronic inflammation.

Structurally, the presence and diversity of the microbiome influence the development and organization of secondary lymphoid organs such as Peyer’s patches and mesenteric lymph nodes—critical sites for the activation and regulation of adaptive immune responses [37]. It is within these environments that the functional maturation of the adaptive immune system occurs, including the clonal expansion and differentiation of T and B lymphocytes in response to a broad range of microbial antigens [47].

A key component of this immune machinery is the development of regulatory T cells (Tregs), which are essential for maintaining immune tolerance and preventing autoimmune or uncontrolled inflammatory reactions [48]. The differentiation and functionality of Tregs are largely dependent on specific signals from the gut microbiome, including metabolites like butyrate, which have potent immunomodulatory effects [36].

Moreover, the microbiome plays a critical role in the induction and maintenance of secretory immunoglobulin A (IgA) in the intestinal mucosa. This immunoglobulin serves as a first-line immune barrier, neutralizing pathogens and selectively shaping the composition of the commensal microbiota, thereby contributing to intestinal homeostasis [46].

However, disruption of this balance, known as dysbiosis, during the early years of life can significantly impair immune maturation. Various studies have linked early-life dysbiosis to an increased incidence of immune-mediated conditions such as allergies, asthma, inflammatory bowel diseases, and autoimmune disorders [47,49,50].

The continuous dialogue between the microbiome and the immune system is not only indispensable for establishing effective immunity but also for maintaining the fine regulation required to prevent inappropriate immune responses, safeguarding health from infancy through adulthood.

### 2.6. Critical Window During Infancy

Early childhood represents a critical developmental window during which the microbiome plays a decisive role in the maturation of the immune system, with repercussions that may persist throughout life [49]. This period, extending from birth to approximately the first three years of life, is marked by heightened biological plasticity and vulnerability, during which key interactions between the host and its microbial community are consolidated [35].

During this phase, the composition, diversity, and stability of the emerging gut microbiota not only reflect the child’s immediate environment but also serve as essential modulators of immune development [51]. A balanced microbiome during this critical stage promotes proper immune training, enabling the distinction between real threats and harmless stimuli. This allows for effective responses against pathogens while maintaining immune tolerance to non-harmful antigens such as food proteins, commensal microbes, and self-antigens [6].

However, various perinatal and environmental factors can disrupt this initial colonization process and thereby influence the individual’s immunological trajectory. These include caesarean delivery, which limits exposure to the maternal vaginal microbiota; formula feeding, which lacks many of the immunomodulatory components present in breast milk; early use of broad-spectrum antibiotics, which can cause persistent dysbiosis; and reduced contact with beneficial environmental microbes due to overly sanitized lifestyles [18]. Such disruptions have been associated with an increased risk of immune-mediated conditions such as allergies, asthma, inflammatory bowel disease (IBD), and other immunological dysfunctions later in life [52].

Current research is focused on elucidating the molecular and cellular mechanisms through which the microbiome shapes the immune system during this critical developmental window [36]. This growing body of knowledge has sparked interest in early-life interventions aimed at favorably modulating the infant microbiome. These include the use of probiotics, defined as live microorganisms with beneficial effects, and prebiotics, which are non-digestible compounds that selectively stimulate the growth of favorable bacteria such as *Bifidobacterium* and *Lactobacillus* [39].

Recognition of this period as a critical window for establishing optimal immune health has significant implications for pediatric and perinatal clinical practice. Designing public health policies and early intervention strategies focused on preserving and promoting a healthy microbiome may prove to be a key tool in reducing the incidence of immune-mediated diseases and improving long-term health outcomes across the life course [21].

#### Communication Mechanisms Between the Microbiome and the Immune System

The relationship between the human microbiome and the immune system is grounded in a highly complex and finely regulated bidirectional communication network that is essential for maintaining host homeostasis. This dynamic interaction not only influences the maturation and functionality of the immune system but also enables precise discrimination between commensal microorganisms and pathogens, allowing for efficient protective responses without compromising tissue integrity or inducing chronic inflammation [53,54].

From the earliest stages of development, the microbiome plays a fundamental educational role by promoting immune tolerance toward symbionts and harmless environmental antigens, while enhancing the immune system’s ability to recognize and eliminate potential threats. This continuous communication is mediated by specific molecular signals that serve as bridges between microbial components and host immune cells, enabling effective and adaptive immunological regulation [34,53,55]. A thorough understanding of these mechanisms is crucial to elucidating the multifactorial origins of chronic inflammatory diseases, autoimmune disorders, and even certain types of cancer. Moreover, it provides a strong theoretical foundation for the development of innovative therapeutic strategies centered on microbiome modulation to restore immune balance and prevent or treat complex diseases [34,56].

### 2.7. Microbial Signaling: MAMPs, PRRs, and TLRs

The immune system has evolved to recognize and respond effectively to microorganisms through highly conserved mechanisms that detect specific molecular structures. This recognition represents the first link in the complex communication network between the host and its microbiome and is fundamental for maintaining immune homeostasis and preventing uncontrolled inflammatory responses. This communication is based on the recognition of microbe-associated molecular patterns (MAMPs), conserved microbial structures detected by specialized receptors of the innate immune system known as pattern recognition receptors (PRRs). Among these, Toll-like receptors (TLRs) stand out as key components of immune surveillance, with well-documented roles in both physiological and pathological contexts [55].

MAMPs are highly conserved structural components of bacteria, viruses, and fungi, such as lipopolysaccharides (LPS), peptidoglycans, flagellin, lipoteichoic acids, and microbial nucleic acids. Unlike classical virulence factors, MAMPs do not necessarily indicate an active infection but are enough to trigger immune responses through PRR-mediated recognition by innate immune cells [57].

PRRs are expressed by immune cells such as macrophages, dendritic cells, epithelial cells, and certain B lymphocytes and are located on the cell membrane, in endosomal compartments, or in the cytoplasm. Among them, TLRs have been extensively studied for their central role in immune surveillance and regulation of tolerance toward the commensal microbiota [58]. Each TLR recognizes a specific class of MAMPs. For instance, TLR4 detects LPS from Gram-negative bacteria, TLR5 recognizes flagellin, and TLR9 binds to unmethylated CpG motifs in bacterial DNA. To date, at least ten functional TLRs have been identified in humans (TLR1–TLR10), each contributing uniquely to immune responses [58]. This specificity allows the immune system to generate tailored responses capable of distinguishing between danger signals and harmless microbial stimuli.

TLR activation triggers intracellular signaling cascades that ultimately activate transcription factors such as NF-κB, IRF3, and IRF7, which drive the expression of pro-inflammatory cytokines, type I interferons, and other immune mediators [59]. However, TLR activation does not always result in overt inflammation; the immunological context and the intensity of stimulation are critical determinants of whether the response is immunostimulatory or tolerogenic [60].

In the intestinal mucosa, where exposure to MAMPs is continuous, TLRs play diverse roles beyond defense. They promote the production of antimicrobial peptides, enhance epithelial tight junction integrity, and stimulate secretory IgA production, thus reinforcing the intestinal barrier without inducing excessive inflammation [61]. This controlled activation is essential for maintaining symbiosis with the commensal microbiota and for preventing inflammatory conditions such as inflammatory bowel disease (IBD). The immune system also employs regulatory mechanisms to limit TLR signaling and prevent exaggerated responses. Proteins such as SIGIRR, IRAK-M, and A20 function as molecular brakes that fine-tune immune activation [62]. This balance is particularly critical in the gut, where the immune system is continuously exposed to a high antigenic load without triggering pathological responses [34].

To TLRs, NOD1 and NOD2 represent cytoplasmic PRRs with complementary functions. These receptors recognize bacterial peptidoglycan fragments and cooperate with TLRs to modulate immune responses [62]. Their relevance has been highlighted in diseases such as Crohn’s disease, where mutations in the NOD2 gene are associated with impaired microbial recognition and dysfunctional inflammatory responses [63].

Together, signaling mediated by MAMPs, PRRs, and TLRs enables the detection and elimination of pathogenic microorganisms while also playing a key role in establishing immune tolerance and maintaining a harmonious relationship with the commensal microbiota. The precision and regulation of these processes largely determine the balance between health and disease within the microbiome–immune axis.

#### 2.7.1. Microbial Metabolites, Tregs and the Intestinal Barrier as Pillars of the Microbiome–Immunity–Homeostasis Axis

A growing body of evidence highlights the pivotal role of microbial-derived metabolites as immunomodulatory mediators at the host–microbiota interface. These bioactive compounds, synthesized by commensal bacteria through fermentation and biochemical transformations, influence both innate and adaptive immune pathways. Among the most studied are short-chain fatty acids (SCFAs), indoles, secondary bile acids, and microbial vitamins, each exerting specific effects on immune cells and epithelial function through receptor-mediated signaling or epigenetic modulation [64,65,66]. Microbial metabolism is therefore not a mere by-product of bacterial fermentation but a fundamental source of signaling molecules that regulate immunological, epithelial, and even neuromodulatory functions, positioning microbial metabolites as key intermediaries in the symbiotic relationship between host and microbiome.

Among these metabolites, SCFAs—mainly acetate, propionate, and butyrate—represent the most relevant immunoregulatory molecules. They are end-products of bacterial fermentation of non-digestible dietary fibers and are found in high concentrations in the colon, where they perform multiple functions [67,68]. Butyrate, for example, is a potent epigenetic modulator that inhibits histone deacetylases (HDACs), thereby promoting the expression of anti-inflammatory genes and enhancing the differentiation of regulatory T cells (Tregs) [69]. Beyond intestinal immunity, SCFAs exert systemic effects, as they can cross the intestinal barrier and reach peripheral tissues such as the lungs and the central nervous system [70]. Acetate, although less potent in epigenetic modulation, stimulates mucin secretion by goblet cells, reinforces the epithelial barrier, and promotes Treg differentiation through activation of G-protein-coupled receptors such as GPR43 [21]. Propionate, for its part, can cross the blood–brain barrier and modulate microglial activity, thus contributing to the regulation of neuroimmune inflammation [71,72]. Collectively, the immunological effects of SCFAs are mediated by their interaction with receptors such as GPR41, GPR43, and GPR109A, expressed on epithelial, immune, and endothelial cells. Activation of these receptors induces IL-10 production, reduces IL-6 and TNF-α, and promotes a more tolerogenic immune profile [73]. In particular, the SCFA–GPR43 axis regulates Treg expansion and macrophage homeostasis, playing a central role in preventing inflammatory and metabolic diseases [74].

The importance of SCFAs in immunoregulation also lies in their ability to promote tolerance and homeostasis through cross-talk with other microbial metabolites. Tryptophan-derived indoles, kynurenic acid, and serotonin influence mucosal immunity by engaging the AhR/IL-22 pathway, which is crucial for maintaining epithelial integrity, inducing antimicrobial peptide synthesis, and promoting tissue repair [75,76]. Indoles also support Treg expansion and the differentiation of group 3 innate lymphoid cells (ILC3s), which are essential for defense against extracellular bacteria at mucosal surfaces [77]. Similarly, secondary bile acids derived from microbial transformation of primary hepatic bile acids exert immunomodulatory roles through the FXR/TGR5 axis, promoting epithelial regeneration and suppressing inflammation [78]. Certain secondary bile acids act as TGR5 agonists, stimulating IL-10 production in dendritic cells and fostering an anti-inflammatory environment [79], while also modulating NLRP3 inflammasome activation, thereby linking bile acids directly to the regulation of intestinal and systemic inflammation [80]. Furthermore, vitamins synthesized by the microbiota—such as biotin, folic acid, and vitamin K—also contribute to immune balance. For instance, biotin-producing strains like *Bifidobacterium adolescentis* have been shown to inhibit the NF-κB transcription factor, while folate promotes Treg survival and function, thus enhancing mucosal tolerance [81,82].

In this context, Tregs emerge as a central component in orchestrating immune tolerance within the microbiome–immunity axis. These cells, crucial for preventing uncontrolled inflammation and autoimmune reactions, are strongly influenced by microbial metabolites such as SCFAs. Butyrate, in particular, enhances Treg differentiation and function via HDAC inhibition and GPR43/GPR109A receptor activation, inducing a transcriptional program that favors the secretion of anti-inflammatory cytokines such as IL-10 and TGF-β [69,73]. Additionally, acetate and propionate modulate dendritic cell function towards a tolerogenic phenotype, indirectly fostering Treg expansion [83,84]. Beyond SCFAs, indoles and folate also reinforce Treg stability and expansion, highlighting the synergy between microbial metabolites and regulatory lymphocyte responses. The importance of Tregs in this framework is underscored by germ-free models, which exhibit reduced Treg populations, impaired tolerance, and increased susceptibility to inflammatory diseases [85]. Thus, the SCFA–Treg axis represents one of the most solid links between microbial metabolism and immune homeostasis.

The interplay between microbial metabolites, Tregs, and epithelial barrier integrity is crucial, as the intestinal barrier represents the first line of defense between the host’s internal milieu and the microbiota-rich lumen. This barrier is not only a physical wall preventing pathogen translocation but also an active immunological organ that integrates signals from the microbiome [53,84]. Its structural components include a monolayer of epithelial cells, a protective mucus layer, tight junction proteins, antimicrobial peptides, and secretory immunoglobulin A (sIgA) [86]. Within this barrier, specialized cells such as enterocytes, goblet cells, Paneth cells, and M cells coordinate microbial sensing via PRRs like TLRs and NLRs [86]. Moderate recognition of microbial-associated molecular patterns (MAMPs) by epithelial PRRs induces basal signaling that promotes mucin secretion, defensin production, and cytokines such as IL-18 and IL-22, without triggering excessive inflammation [87]. IL-22, secreted by ILC3s and Th17 cells, is central to this protective response, as it stimulates epithelial regeneration, antimicrobial peptide synthesis, and tight junction strengthening [88,89].

SCFAs, particularly butyrate, directly enhance epithelial integrity by upregulating tight junction proteins through the HIF-1α pathway [90], while acetate promotes mucin production and synergizes with sIgA to strengthen the mucus layer [21,91]. Paneth cells secrete antimicrobial peptides like α-defensins and lysozyme to control microbial proliferation near the epithelium, and their dysfunction has been linked to Crohn’s disease [92]. Goblet cells, through mucin secretion, establish a physical barrier that shapes the microbiota itself, as shown by the association of *Akkermansia muciniphila* with epithelial health [93]. Dendritic cells and macrophages in the lamina propria sample luminal antigens and promote Treg differentiation in mesenteric lymph nodes under homeostatic conditions [83,94]. In parallel, sIgA coats commensal bacteria, preventing epithelial adhesion and neutralizing pathogens while favoring colonization by beneficial taxa [95]. Loss of these coordinated interactions—due to dysbiosis, metabolite deficiency, or epithelial disruption—triggers increased permeability (“leaky gut”), allowing microbial antigens to enter the bloodstream and fuel systemic inflammation and autoimmunity [96].

Altogether, the integration of SCFAs, Treg modulation, and epithelial barrier integrity defines the microbiome–immunity–homeostasis axis. This axis functions as a feedback loop: microbial metabolites promote Treg induction and epithelial health, while the immune system and barrier mechanisms shape microbial composition and metabolite production. When this balance is preserved, it ensures tolerance, pathogen defense, and systemic equilibrium. Conversely, its disruption by genetic, dietary, or environmental factors leads to dysbiosis, chronic inflammation, and immune-mediated diseases ranging from IBD and metabolic syndrome to neuropsychiatric and autoimmune disorders [84,85,97,98,99,100,101,102]. Thus, understanding the interplay of microbial metabolites, Treg biology, and barrier integrity not only clarifies the mechanisms of host–microbiome symbiosis but also opens therapeutic avenues based on probiotics, prebiotics, postbiotics, and fecal microbiota transplantation to restore homeostasis and improve clinical outcomes.

#### 2.7.2. How Microbiome–Immune System Synergy Maintains Physiological Balance?

The physiological balance of the human body depends largely on the synergistic interaction between the intestinal microbiome and the host immune system. This symbiotic relationship not only enables constant immune surveillance but also ensures finely tuned responses against pathogens while maintaining tolerance toward commensal microorganisms and dietary antigens. In this way, intestinal homeostasis emerges as the result of a complex, bidirectional molecular dialogue in which the microbiome and immune system co-regulate to preserve epithelial integrity, barrier function, and a balanced immune response.

Recent research has shown that the microbiome is not merely a passive barrier against external agents but also an active modulator of immunity. A central element in this interaction is the role of microbial metabolites, particularly short-chain fatty acids (SCFAs). These fermentation products of dietary fibers directly influence the differentiation and function of immune cells, especially regulatory T cells (Tregs), which are key for inducing immune tolerance and preventing exaggerated inflammatory responses [103].

Moreover, communication between the microbiome and the immune system is mediated by the regulated activation of pattern recognition receptors (PRRs), including Toll-like receptors (TLRs) and NOD-like receptors (NLRs). Their activation enables the immune system to detect specific microbial signals and modulate its responses accordingly. As a result, anti-inflammatory cytokines such as IL-10 and TGF-β are produced, alongside antimicrobial peptides, contributing to intestinal barrier strengthening and limiting local inflammation [63].

The intestinal epithelium also plays a critical role in this functional synergy. Far from being a passive barrier, epithelial cells actively respond to microbial signals by secreting mucus, renewing the mucosal layer, and producing immunomodulatory factors. Epithelial integrity is essential to prevent bacterial translocation and uncontrolled immune activation, which could lead to systemic inflammatory processes [73].

Notably, this microbiome–immune cooperation is dynamic and adaptive, particularly during early life. Gut colonization in the first days after birth is crucial for immune system maturation, directly influencing susceptibility to allergic and autoimmune diseases later in life. Early exposure to a diverse microbiome promotes the development of tolerant and functional immunity, as demonstrated in multiple studies on neonates [104].

Furthermore, this synergy extends beyond the intestine and impacts systemic immunity. Immune cells trained by microbial signals can migrate to other tissues and continue performing immune surveillance functions. This interconnection explains how disruptions in gut microbial composition can trigger distant effects associated with autoimmune, metabolic, and even neurological diseases [105].

Maintaining physiological balance thus requires a continuous and harmonious dialogue between the microbiome and the immune system. This interaction not only ensures protection against pathogens but also supports immune tolerance, preserves tissue integrity, and regulates inflammation. Loss of this synergy, due to dysbiosis, environmental factors, or genetic predisposition, may result in chronic inflammatory diseases, underscoring the clinical and therapeutic importance of preserving and modulating this interaction [106].

#### 2.7.3. Response to Pathogens vs. Immune Tolerance

The immune system faces a fundamental challenge: it must recognize and eliminate potentially harmful pathogens while avoiding inflammatory responses against commensal microorganisms or harmless dietary antigens. This functional duality is essential for maintaining intestinal and systemic homeostasis, and it relies on a complex network of regulatory mechanisms that allow the immune system to discriminate between real threats and benign stimuli, thus preventing tissue damage and autoimmune disorders.

Defense against pathogens begins with the detection of pathogen-associated molecular patterns (PAMPs) through pattern recognition receptors such as TLRs and NLRs. This initial activation triggers intracellular signaling cascades that culminate in the production of pro-inflammatory cytokines (including IL-1β, TNF-α, and IL-6) and the recruitment of innate and adaptive immune cells, such as macrophages, neutrophils, dendritic cells, and effector T cells [89]. The result is a coordinated immune response aimed at rapid and efficient elimination of the invading pathogen.

However, the same immune machinery that fights infections must simultaneously maintain tolerance to the resident microbiota, which plays essential roles in digestion, metabolite production, and competitive defense against pathogens. This immune tolerance is primarily mediated by regulatory T cells (Tregs), whose activity is promoted by anti-inflammatory cytokines like IL-10 and TGF-β, creating a controlled immune environment in the intestinal mucosa [107].

A key player in this regulation is the mucosal dendritic cell, which can present antigens in a context-dependent manner. When sampling commensal or dietary antigens in the absence of danger signals, these cells promote tolerance induction by stimulating Treg expansion and enhancing secretory IgA (sIgA) production. This immunoglobulin acts as a non-inflammatory barrier, limiting direct bacterial–epithelial interaction [90].

Microbiome-derived metabolites, particularly SCFAs, play a central role in promoting tolerance. Butyrate, for example, enhances Treg suppressive function and helps maintain epithelial barrier integrity, thereby reducing susceptibility to chronic inflammation [92].

This delicate balance between pro-inflammatory and tolerogenic signals depends heavily on the composition and metabolic activity of the intestinal microbiome. When this equilibrium is disrupted (by dysbiosis, infection, medication, or epithelial damage), the immune system may become inappropriately activated, triggering chronic inflammation that contributes to conditions such as ulcerative colitis, Crohn’s disease, food allergies, and autoimmune disorders [91].

In these scenarios, uncontrolled immune activation and loss of tolerance fuel an inflammatory cycle that perpetuates local tissue damage and may have systemic consequences. In contrast, while the immune response to pathogens involves the formation of immunological memory for future protection, immune tolerance is an ongoing, adaptable process that must constantly adjust to changes in the microbiota and dietary inputs [93].

An additional dimension to this regulation has been identified in the neuroimmune interface. The enteric nervous system, through neurotransmitters and neuromodulators, participates in the modulation of intestinal immunity. These signals can influence both epithelial permeability and immune cell activity, demonstrating that immune equilibrium is also subject to cross-talk between the nervous and immune systems [94].

#### 2.7.4. Dysbiosis and Its Immunological Consequences

Dysbiosis is defined as an imbalance in the composition and function of the microbiome, particularly within the gastrointestinal tract, that disrupts the symbiotic relationship between the host and its microbial communities. This phenomenon plays a critical role in immune system dysregulation, affecting both innate and adaptive immunity, and is associated with the onset and perpetuation of numerous inflammatory, autoimmune, and metabolic diseases.

Under healthy conditions, the intestinal microbiota performs essential functions in the maturation and regulation of the immune system. These include the induction of immune tolerance toward commensal antigens and protection against pathogens through niche competition and the production of beneficial metabolites. However, when this balance is disrupted, the loss of key bacterial species, alongside the expansion of potentially pathogenic microorganisms, leads to a pro-inflammatory state characteristic of dysbiosis [83].

One of the most significant consequences of dysbiosis is the compromise of the intestinal epithelial barrier, a key component of immune defense. Disruption of this barrier increases intestinal permeability—commonly referred to as “leaky gut”—which facilitates the translocation of bacteria and microbial products such as lipopolysaccharides (LPS) into the systemic circulation. This in turn triggers a widespread inflammatory response that can impact distant tissues and organs [95].

On one hand, it alters the proportion and functionality of immune cells, including a reduction in regulatory T cells (Tregs), crucial for maintaining immune tolerance, and an increase in pro-inflammatory Th17 cells, which produce IL-17, a key cytokine in chronic inflammation [96]. This cellular imbalance fosters an inflammatory environment conducive to the development of autoimmune and inflammatory diseases. Additionally, dysbiosis impairs the production of critical microbial metabolites such as short-chain fatty acids (SCFAs), particularly butyrate and propionate, which are vital for maintaining epithelial integrity and inducing Tregs. Decreased SCFA concentrations have been linked to intestinal barrier dysfunction and heightened inflammation [85].

Moreover, an impoverished and imbalanced microbiota undermines mucosal immunity, reducing the production of antimicrobial peptides and secretory IgA antibodies, both of which are essential for protecting mucosal surfaces. This increases susceptibility to bacterial, viral, and fungal infections [84].

The persistent stimulation of pattern recognition receptors (PRRs) by microbial products abnormally activates the innate immune system, perpetuating chronic inflammation. This mechanism has been implicated in a variety of conditions, including inflammatory bowel diseases, metabolic syndrome, and neuroinflammatory disorders such as multiple sclerosis and Alzheimer’s disease, underscoring the systemic immunological consequences of dysbiosis and its role in the gut–brain axis [97] (Table 1).

The composition of the intestinal microbiome has been shown to influence the effectiveness of immune-based therapies, particularly cancer immunotherapy. Specific microbial configurations have been associated with enhanced responses to immune checkpoint inhibitors, opening the door to personalized treatment strategies based on microbiome profiling [98].

#### 2.7.5. Microbiome Alterations and Inflammatory Diseases

Among the most significant clinical manifestations of dysbiosis are inflammatory bowel diseases (IBD), particularly Crohn’s disease (CD) and ulcerative colitis (UC). These chronic disorders are characterized by persistent inflammation of the gastrointestinal tract, with a multifactorial etiology involving genetic, environmental, and immunological components. However, intestinal dysbiosis has emerged as a central factor in their pathogenesis (Table 1).

Genomic and metagenomic sequencing studies have shown that patients with IBD exhibit a marked reduction in bacterial diversity, particularly among beneficial members of the Firmicutes phylum, such as Faecalibacterium prausnitzii, a well-known anti-inflammatory bacterium and butyrate producer, a short-chain fatty acid essential for maintaining intestinal mucosal integrity [108]. In parallel, an increase in potentially pathogenic bacteria from the Proteobacteria phylum, especially Enterobacteriaceae, has been documented. These bacteria contribute to the inflammatory process through the production of lipopolysaccharide (LPS) and other pro-inflammatory molecules [109].

These microbial imbalances trigger dysregulated activation of both innate and adaptive immune responses. The abnormal interaction between a disturbed microbiota and pattern recognition receptors (PRRs), including Toll-like receptors (TLRs) and NOD-like receptors (NLRs), promotes excessive secretion of pro-inflammatory cytokines such as IL-1β, TNF-α, and IL-6, along with chemokines, fueling a chronic inflammatory loop that progressively damages the intestinal mucosa [110].

At the immunological level, there is a clear imbalance in T cell regulation, characterized by a reduction in regulatory T cells (Tregs) (which impairs inflammation control) and an expansion of Th17 cells, which exacerbate inflammation via secretion of IL-17 and other cytokines [111].

Furthermore, increased intestinal permeability, directly resulting from dysbiosis and epithelial damage, facilitates the translocation of luminal bacteria and antigens into the submucosal tissue. This translocation triggers local and systemic immune responses, further perpetuating disease progression [112].

The consequences of these alterations are not limited to the gastrointestinal tract. It has been demonstrated that dysbiosis in IBD can contribute to systemic inflammation and affect extraintestinal organs, increasing the risk of arthritis, dermatitis, and hepatic diseases [28].

In response to this complex scenario, therapeutic strategies aimed at restoring microbial homeostasis, such as fecal microbiota transplantation (FMT) and the use of specific probiotics, have shown promising results in inducing remission and controlling IBD symptoms. Nevertheless, the high interindividual variability of the microbiome and its complex interactions with the immune system demand personalized approaches to optimize therapeutic efficacy [113].

#### 2.7.6. Dysbiosis and Immune-Mediated, Neurological, and Infectious Diseases

##### Systemic Lupus Erythematosus

Systemic Lupus Erythematosus (SLE) is a complex systemic autoimmune disease characterized by the production of autoantibodies and multi-organ damage. SLE is both influenced by and contributes to gut microbiome dysbiosis. Altered microbial composition can trigger immune imbalance, promoting autoantibody production and systemic inflammation, while the chronic inflammation characteristic of SLE further disrupts beneficial microbial communities, creating a feedback loop that sustains disease progression [100]. Numerous studies have revealed significant alterations in the composition of the gut microbiome in patients with SLE, including a reduction in beneficial commensal bacteria such as *Firmicutes*, *Bifidobacterium*, *Faecalibacterium prausnitzii*, and *Lactobacillus*, and an increase in potentially pathogenic bacteria like *Ruminococcus gnavus* and *Enterococcus gallinarum* [114,115]. These changes not only reflect microbial imbalance but are also associated with disease activity and the presence of autoantibodies, suggesting a direct role of the microbiome in SLE pathogenesis [116]. Moreover, functional shifts have been observed in the microbiome of SLE patients, such as decreased production of short-chain fatty acids (SCFAs) and increased pro-inflammatory metabolites like lipopolysaccharide (LPS) and trimethylamine N-oxide (TMAO), which may contribute to systemic inflammation and immune dysregulation characteristic of the disease [117].

From an immunological perspective, the gut microbiota modulates the balance between regulatory T cells (Tregs) and pro-inflammatory T helper 17 (Th17) cells. In SLE, an expansion of Th17 cells and a reduction in Tregs have been reported, possibly influenced by alterations in microbiome composition [114]. Furthermore, hyperactivation of inflammatory pathways such as Toll-like receptor 4 (TLR4) signaling in response to microbial components like LPS has been identified in lupus, contributing to systemic immune activation [118].

Animal studies have demonstrated that fecal microbiota transplantation (FMT) from SLE patients into germ-free mice induces hallmark disease features, including autoantibody production and renal inflammation, supporting a causal role for the microbiome in SLE pathogenesis [115]. Additionally, the concept of molecular mimicry—where microbial epitopes resemble self-antigens—has been implicated in SLE, exemplified by *R. gnavus* antigens cross-reacting with lupus nephritis autoantibodies [114]. These findings underscore the gut microbiome as a critical environmental cofactor interacting with genetic predisposition to initiate and sustain autoimmunity in SLE.

##### Antiphospholipid Syndrome

The antiphospholipid syndrome (APS) may be influenced by the gut microbiome through molecular mimicry, in which commensal bacteria activate autoreactive cells that induce the production of antiphospholipid autoantibodies [63]. It is influenced by and contributes to gut microbiome dysbiosis. Molecular mimicry, where gut microbial proteins resemble host autoantigens, may trigger an autoimmune response, while increased intestinal permeability promotes systemic inflammation and thrombotic events. These interactions suggest novel therapeutic strategies complementary to anticoagulation [119]. Moreover, dysbiosis may increase intestinal permeability, promoting systemic inflammation and thrombotic events [63,73]. Although not all studies have shown marked microbial differences in APS [104], it has been proposed that the functional impact of the microbiome on coagulation and inflammation may be more relevant than its specific taxonomic composition [63,73,104]. This connection suggests novel therapeutic strategies complementary to anticoagulation, such as the use of probiotics, personalized diets, or fecal microbiota transplantation [103,105]. It may also contribute to a better understanding of the coexistence of APS with systemic lupus erythematosus, given the clinical overlap between both conditions [106].

##### Multiple Sclerosis

Multiple Sclerosis (MS) is an autoimmune disease of the central nervous system characterized by demyelination and chronic inflammation. Multiple sclerosis (MS) is both influenced by and contributes to gut microbiome dysbiosis. Altered microbial composition can trigger immune imbalance, promoting Th17-cell differentiation and inhibiting regulatory T cells (Tregs), while chronic inflammation characteristic of MS further disrupts beneficial microbial communities, creating a feedback loop that sustains disease progression. Recent studies have confirmed these interactions, highlighting the critical role of the gut microbiome in MS pathogenesis and suggesting potential therapeutic avenues targeting microbial modulation [120]. Studies of MS patients have consistently identified alterations in gut microbiome composition, including a reduction in SCFA-producing bacteria such as Faecalibacterium prausnitzii and an increase in pro-inflammatory taxa such as Akkermansia muciniphila, Methanobrevibacter smithii, and Desulfovibrio [103,121]. These microbial shifts contribute to intestinal dysbiosis and systemic immune activation by promoting Th17-cell differentiation and inhibiting regulatory T cells (Tregs), thus favoring a pro-inflammatory environment [122].

Furthermore, the gut–brain axis plays a crucial role in the bidirectional communication between the intestine and central nervous system, influencing neuroinflammation through immune, hormonal, and neural signaling pathways [123]. Experimental models have shown that fecal microbiota transplantation (FMT) from MS patients exacerbates disease severity in mice, whereas restoration of beneficial bacteria attenuates disease progression, underscoring the potential of microbiome-targeted therapeutics [121,124].

##### Rheumatoid Arthritis

Rheumatoid arthritis (RA) is a chronic autoimmune disease primarily affecting the joints. Several studies have reported gut dysbiosis in RA patients, characterized by an increased abundance of bacteria such as *Prevotella copri*, which has been linked to the emergence of autoantibodies and immune system activation [125,126]. This dysbiosis may compromise intestinal permeability, facilitating the translocation of bacterial products such as lipopolysaccharide (LPS) into the bloodstream, thereby triggering systemic inflammatory responses [127]. Moreover, the reduction in short-chain fatty acids (SCFAs), particularly butyrate, may impair intestinal barrier integrity and negatively modulate immune responses, promoting chronic joint inflammation [82,128]. The microbiome also influences T and B cell differentiation, enhancing activation of autoreactive lymphocytes and production of autoantibodies such as rheumatoid factor (RF) and anti-citrullinated protein antibodies (ACPAs) [129].

##### Colorectal Cancer

The gut microbiota is implicated in the progression of colorectal cancer (CRC) through its influence on inflammation and antitumor immune responses [130,131]. It is influenced by and contributes to gut microbiome dysbiosis. Dysbiotic microbiota can promote tumor initiation and progression through inflammation, genotoxic metabolites, and immune dysregulation, while the evolving tumor environment further selects cancer-associated microbes, reinforcing a pro-carcinogenic microbial community and sustaining the disease. Recent reviews emphasize the microbiome’s dual role in CRC pathogenesis and its potential as a biomarker and therapeutic target [84,132].

While total microbial diversity may not significantly change, shifts in the abundance of major phyla—such as increased Bacteroidetes, Firmicutes, and Fusobacteria, and decreased Proteobacteria—have been observed [84,133]. Oncogenic bacterial species are associated with advanced cancer stages, lymph node involvement, and poor prognosis [134,135]. The microbiome may serve as a biomarker and an early diagnostic tool [108,130]. Microbial modulation through probiotics and symbiotics shows promise in enhancing immune responses and slowing tumor progression. Moreover, specific microbiome profiles have been linked to improved responses to immunotherapy, paving the way for personalized medicine approaches [103].

##### Allergic Diseases (Asthma and Atopic Dermatitis)

Allergic diseases, including asthma and atopic dermatitis, are influenced by gut microbiome composition. Dysbiosis of the early-life gut microbiome predisposes individuals to allergic diseases by impairing immune maturation and SCFA production, while allergic inflammation and Th2 skewing further disrupt beneficial microbial communities, reinforcing the atopic phenotype [136]. Reduced microbial diversity in early life has been associated with increased risk of developing allergic conditions [96]. The presence of beneficial SCFA-producing bacteria such as *Bifidobacterium* and *Faecalibacterium* correlates with a balanced immune response, whereas their reduction may promote polarization towards Th2 cells, which are characteristic of allergic diseases [137]. Furthermore, the gut microbiome modulates immune system maturation during early life, and disruption of this process can have lasting impacts on susceptibility to allergic disorders [87].

##### Alzheimer’s Disease

Alzheimer’s disease (AD), the leading cause of dementia, is characterized by β-amyloid plaques and tau neurofibrillary tangles in the brain. Gut dysbiosis promotes neuroinflammation, amyloid and tau pathology, and blood–brain barrier disruption in Alzheimer’s disease, while AD-related neurodegeneration further worsens microbial imbalance, establishing a bidirectional feedback loop [138]. Gut dysbiosis in AD patients is marked by increased pro-inflammatory bacteria such as *Escherichia*/*Shigella* and decreased anti-inflammatory species like *Bifidobacterium* and *Faecalibacterium prausnitzii* [102,139]. These microbial alterations are associated with increased intestinal permeability and translocation of pro-inflammatory metabolites such as LPS, which can cross the blood–brain barrier and activate microglia, promoting neuroinflammation and neurodegeneration [82]. The gut–brain–microbiota axis mediates bidirectional communication between the intestine and brain, modulating immune, hormonal, and metabolic signals; disruption of this axis contributes to cognitive decline and disease progression [140]. SCFAs, particularly butyrate, have demonstrated neuroprotective effects, and their reduction in AD may exacerbate pathology. Animal studies show that microbiota manipulation via FMT or probiotics can reduce β-amyloid accumulation and improve cognition, suggesting a modulatory microbiome role in AD [141,142].

##### Parkinson’s Disease (PD)

Parkinson’s Disease (PD) is a neurodegenerative disorder marked by progressive loss of dopaminergic neurons in the substantia nigra. The gut microbiota of PD patients exhibits significant alterations, including a reduction in butyrate-producing bacteria such as *Faecalibacterium prausnitzii* and an increase in pro-inflammatory taxa like Enterobacteriaceae and Lactobacillus [134,143]. This loss of fermentative commensals impairs intestinal production of anti-inflammatory metabolites such as butyrate, which maintains epithelial barrier integrity, modulates immune responses, and regulates neuroprotective gene expression [108]. Reduced butyrate levels may increase intestinal permeability, facilitating translocation of LPS and other bacterial toxins into the bloodstream and central nervous system [144]. Additionally, α-synuclein aggregation, a hallmark of PD, may originate in the gut and propagate to the brain via the vagus nerve, driven by microbiota-derived pro-inflammatory signals [145]. Fecal microbiota transplantation (FMT) from PD patients into animal models replicates motor and neuroinflammatory symptoms, supporting a functional role of the microbiome in disease progression [143].

##### Autism Spectrum Disorder

Numerous studies have shown that children with ASD present a distinct gut microbiota, characterized by a reduction in beneficial genera such as *Prevotella*, *Coprococcus*, and Bacteroides, which are key producers of short-chain fatty acids (SCFAs) and essential for intestinal homeostasis [134,137,143]. Bifidobacterium, important for immunomodulation and neurotransmitter synthesis, has also decreased [103,143]. These alterations have been linked to both gastrointestinal and behavioral symptoms in ASD [108,144]. Recent studies have identified altered tryptophan-derived metabolites, such as quinolinic acid, which are implicated in emotional and sensory processing [139,145]. Gut dysbiosis in ASD may contribute to neurodevelopmental symptoms through altered SCFA production and immune and metabolic disruption, while ASD-related gastrointestinal and behavioral traits can further perturb the microbiota, forming a bidirectional feedback loop [146]. These microbial differences appear to be associated with ASD itself rather than diet alone [108,137]. In response, therapeutic approaches such as probiotics and fecal microbiota transplantation are being developed, with preliminary results showing promise [102,145]. However, further research is needed to establish safe and effective treatments.

##### Attention Deficit Hyperactivity Disorder (ADHD)

Children with Attention Deficit Hyperactivity Disorder (ADHD) have been shown to exhibit an altered gut microbial composition compared to healthy individuals, suggesting a dysbiosis state with pathophysiological implications. A notable reduction in *Faecalibacterium*, a butyrate-producing bacterium with anti-inflammatory properties, has been correlated with greater ADHD symptom severity [127,142]. Elevated levels of lipopolysaccharide-binding protein (LBP) have also been reported, indicating possible gut barrier dysfunction and systemic inflammatory activation [147,148]. Additionally, unmedicated children show a lower abundance of Dialister, which appears to be restored following pharmacological treatment [149,150]. In contrast, *Bifidobacterium* levels are elevated in ADHD but tend to decrease after micronutrient supplementation, suggesting diet-driven dynamics [148,149]. While these findings are preliminary, they point to a modulatory role of the gut microbiome in ADHD neurobiology and open innovative therapeutic avenues, such as the use of probiotics, prebiotics, or personalized diets [125,128,129].

##### Schizophrenia

Schizophrenia is associated with gut microbiome alterations, including reduced microbial diversity and a distinct bacterial profile compared to healthy controls [99]. This dysbiosis favors the proliferation of pro-inflammatory bacteria, compromises intestinal barrier function, and facilitates LPS translocation into systemic circulation, inducing neuroinflammation [100]. Altered synthesis of neurotransmitters such as GABA, serotonin, and dopamine has also been linked to microbial changes in schizophrenia [101]. Furthermore, elevated pro-inflammatory cytokines, including IL-6 and TNF-α, reinforce gut–immune axis involvement in disease pathology [102]. Animal models reproduce schizophrenia-like behaviors following FMT from patients, supporting a causal dysbiosis role [102]. Gut microbiome dysbiosis contributes to schizophrenia via neuroinflammation, altered neurotransmitter production, and vagus-mediated signaling, while the disorder itself and its treatments exacerbate microbial imbalance, constituting a bidirectional feedback loop [151]. These findings highlight microbiome modulation therapies—probiotics, diet, or FMT—as potential clinical strategies [103].

##### Depression

Recent studies indicate that individuals with depression exhibit alterations in the gut microbiota, with a reduction in anti-inflammatory bacteria and an increase in pro-inflammatory species [101,102,152]. There is a decreased abundance of butyrate-producing bacteria such as *Faecalibacterium prausnitzii* and *Bifidobacterium* spp., which play critical roles in maintaining intestinal barrier integrity and immune regulation [103,153]. Simultaneously, there is an increase in genera such as Desulfovibrio and *Escherichia*/*Shigella*, which are associated with systemic inflammation [101,152]. These alterations are linked to elevated levels of IL-6, TNF-α, and C-reactive protein, suggesting an inflammatory component to depression [153]. The gut–brain axis mediates this interaction, whereby microbial metabolites, such as short-chain fatty acids and tryptophan derivatives, modulate neurotransmission [154,155,156]. Gut dysbiosis contributes to depression by promoting systemic inflammation, neurotransmitter imbalances, and gut–brain axis disruptions, while depressive pathology further amplifies microbial imbalance, establishing a bidirectional feedback loop [157]. Probiotic strains such as *Lactobacillus* and *Bifidobacterium* have shown potential in alleviating depressive symptoms; however, more clinical studies are needed to establish effective protocols and clarify underlying mechanisms [152,153].

##### Anxiety

The gut microbiota also plays a role in anxiety disorders. Dysbiosis, such as that induced by antibiotic use, may promote the growth of bacteria like Klebsiella *oxytoca*, leading to anxiety and colitis in animal models through neuroinflammation and increased intestinal permeability [158,159]. The microbiota modulates key neurodevelopmental processes, including blood–brain barrier formation, microglial maturation, and neurogenesis [63,160]. Dysbiosis increases systemic inflammation, alters neurotransmitter levels, and impairs synaptic plasticity, favoring anxious behavior [161,162]. Chronic stress affects the microbiome and activates the HPA axis, increasing cortisol levels and perpetuating both intestinal and cerebral inflammation [163,164]. Germ-free animals exhibit cognitive and stress-related abnormalities that can be reversed through microbial recolonization [165]. In humans, probiotics such as *Lactobacillus helveticus* and *Bifidobacterium longum* have been shown to reduce anxiety symptoms [165,166,167]. Prebiotics, like inulin, also show promise [168], though long-term studies are still needed.

##### Infectious Diseases

The microbiota acts as a protective barrier by competing for ecological niches and nutrients and producing antimicrobial metabolites. However, dysbiosis weakens this defense, facilitating colonization by opportunistic pathogens. For instance, in recurrent *Clostridioides difficile* infections, antibiotic use disrupts the microbiota, reduces colonization resistance, and permits pathogen overgrowth, resulting in severe diarrhea and colitis [103,109]. Microbial composition also influences immune responses to respiratory and gastrointestinal viruses. An altered microbiota may reduce vaccine efficacy and antiviral defense, increasing vulnerability to infections such as influenza and SARS-CoV-2 [110,148]. These findings suggest that microbiome modulation through probiotics, prebiotics, or fecal transplants represents a promising therapeutic strategy to restore immune function and enhance infection resistance [111,156].

##### Tuberculosis

Alterations in both the pulmonary and intestinal microbiota play a key role in pulmonary tuberculosis (TB). Respiratory tract dysbiosis is associated with active TB and impacts host immune responses [28,112,113]. Decreased microbial diversity and loss of commensal species, along with increased abundance of pathogenic genera such as *Streptococcus* and *Veillonella*, have been reported in TB patients [28,102,139]. These microbial signatures may serve as biomarkers for diagnosis and prognosis [114,115]. The gut–lung microbiome axis and its interaction with the immune system influence pulmonary inflammation and disease progression [114,116]. Anti-tuberculosis treatments cause intestinal and respiratory dysbiosis [117,139], prompting investigations into complementary therapies using probiotics and prebiotics to restore microbial balance, support immunity, and improve clinical outcomes [115,116,117]. Although evidence is still preliminary, such interventions may reduce inflammation and shorten treatment duration, but broader studies are required.

##### Human Immunodeficiency Virus (HIV)

HIV infection profoundly alters the gut microbiota, with increased microbial diversity and predominance of genera such as *Prevotella*, a pattern that persists even under antiretroviral therapy [82,140,141]. This dysbiosis is linked to chronic inflammation, immune dysfunction, and impaired CD4+ T cell recovery [106,142]. It has also been associated with increased risk of cardiovascular disease and cancer [169,170]. The reduction in SCFA-producing bacteria such as *Faecalibacterium prausnitzii* may contribute to persistent inflammation. Additionally, specific microbial configurations are associated with better immune recovery post-ART [171]. Fecal microbiota transplantation and probiotic therapies have shown promise in restoring eubiosis and reducing inflammation, although robust evidence of their long-term efficacy is still lacking [73,140]. Understanding this interaction could lead to improved quality of life for people living with HIV [106].

**Table 1:** Relationship between microbial dysbiosis and chronic diseases: Experimental and observational evidence.

Disease	Type of Study	Microbial Alterations	Immunological or Systemic Effects	Reference
**Alzheimer’s Disease**	Experimental, systematic review, and observational	Decrease in *Firmicutes*, increase in *Proteobacteria* and *Bacteroidetes*; increase in secondary bile acids; alterations in neuroactive metabolites such as SCFAs	Increased β-amyloid plaques, activation of γ-secretase, systemic inflammation; blood biomarkers	[109,110,111,112].
**Parkinson’s Disease**	Systematic, narrative, experimental, and human studies	↑ *Lactobacillus*, *Akkermansia*, *Bifidobacterium*; ↓ *Lachnospiraceae*, *Faecalibacterium*; ↓ SCFAs; ↑ pro-inflammatory cytokines	Neuroinflammation, gut–brain axis dysfunction, motor symptoms linked to dysbiosis	[28,113,114,115].
**Colorectal Cancer**	Review and observational	Decrease in *Bacteroidetes*; increase in *Firmicutes*; structural alteration of microbiome in tumor tissue vs. intestinal lumen	Promotes invasion, metastasis, tumor inflammation, and response to chemotherapy	[123,124].
**Autism Spectrum Disorder (ASD)**	Observational, metagenomic, functional, and reviews	↑ *Lachnospiraceae*, *Clostridiales*, *Veillonella*, *Alistipes*, *Candida*; ↓ *Faecalibacterium*, *Prevotella*, *Coprococcus*; ↓ GABA, melatonin, butyrate-related genes	Dysbiosis associated with GI and neurological symptoms; diagnostic prediction via microbiome	[155,156,158,159].
**HIV/AIDS**	Observational, review, experimental	↑ *Proteobacteria*, *Prevotella*; ↓ *Bacteroides*, *Firmicutes*, diversity; persistent dysbiosis post-ART	Chronic immune activation, Th17 cell loss, GALT disruption, systemic inflammation	[162,163,164,165].
**Schizophrenia**	Case-control, cross-sectional, cohort	↑ *Collinsella*, *Lactobacillus*, ↓ *Faecalibacterium*, *Anaerostipes*; neuroactive metabolite alterations	Microbiota alteration associated with symptoms and metabolic syndrome; potential biomarker	[142,169,170].
**Tuberculosis**	Observational, experimental, review	↓ *Firmicutes*, *Bifidobacterium*, *Lactobacillus*; ↑ *Proteobacteria*, *Enterobacteriaceae*	Intestinal and pulmonary dysbiosis associated with inflammation, disease progression, and severity	[130,131,168].
**Anxiety**	Experimental, review, clinical	↓ *Lactobacillus*, *Bifidobacterium*, *Prevotella*; ↑ *Proteobacteria*	Dysbiosis alters neurotransmitters like GABA, triggers inflammation and anxiety; improves with probiotics	[152,153,171].
**Antiphospholipid Syndrome (APS)**	Experimental, observational, review	↓ *Lactobacillus*, *Firmicutes*; ↑ *Enterobacteriaceae*, *Bacteroides*	Immune activation, production of antiphospholipid antibodies, thrombosis	[63,160,161,165,167].
**Asthma/Eczema**	Experimental, observational, review	Dysbiosis in *Firmicutes*, *Bacteroidetes*, *Proteobacteria*, *S. aureus*; ↓ *Prevotella*, *Lactobacillus*	Associated with respiratory and dermal inflammation; severity modulated by microbiota	[84,133,154].
**Systemic Lupus Erythematosus (SLE)**	Observational, experimental, systematic reviews	↓ *Firmicutes*, *Lactobacillus*, *Clostridiales*; ↑ *Bacteroidetes*, *Proteobacteria*, *Ruminococcus gnavus*, *Enterococcus*; persistent dysbiosis.	B and T cell activation, ↑ IL-6, IFN-γ, TNF-α, loss of immune tolerance, multi-organ damage	[82,102,139,140,141,145].
**Crohn’s Disease/Ulcerative Colitis (IBD)**	Clinical, experimental, and systematic review studies	↓ *Faecalibacterium prausnitzii*, ↓ *Akkermansia*, ↑ *Escherichia coli*, ↓ *Clostridium leptum*, general dysbiosis	Reduced SCFA, increased pro-inflammatory cytokines, intestinal barrier disruption, exacerbated immune response	[108,134,143,144],
**Irritable Bowel Syndrome (IBS)**	Clinical studies and reviews	↓ *Lactobacillus*, *Bifidobacterium*, ↑ *Enterobacteriaceae*	Visceral hypersensitivity; low-grade inflammation	[129]
**COPD (Chronic Obstructive Pulmonary Disease)**	Clinical and review	↓ Diversity; ↑ *Haemophilus*, *Moraxella*	Chronic inflammation; activation of macrophages and neutrophils	[137]

**↑** The population increases, **↓** The population decreases.

#### 2.7.7. Role of the Microbiome in Chronic Diseases: Obesity, Diabetes, and Cancer

In recent years, growing evidence has positioned the gut microbiome as a central player in the pathophysiology of various non-communicable chronic diseases, including obesity, type 2 diabetes, and different types of cancer. These conditions share a common background characterized by low-grade chronic inflammation, in which dysbiosis plays a key role in perpetuating inflammatory and dysregulated metabolic processes. In this context, the study of microbiota-immune system interactions emerges as a crucial axis for understanding the biological basis of these diseases and for designing more effective therapeutic strategies.

In obesity, numerous studies have documented substantial differences in the composition and function of the gut microbiome between overweight individuals and those with a normal body mass index. Notably, a decrease in microbial diversity and a relative increase in the Firmicutes-to-Bacteroidetes ratio have been observed. This imbalance favors enhanced energy harvest from the diet and promotes lipid storage, thus contributing to the development and maintenance of the obesogenic state [121]. Furthermore, dysbiosis induces increased intestinal permeability, facilitating the translocation of bacterial lipopolysaccharides (LPS) into the bloodstream. This leads to a systemic inflammatory response known as metabolic endotoxemia, which interferes with insulin signaling and promotes the onset of insulin resistance, a key phenomenon in the progression to type 2 diabetes [103].

In type 2 diabetes, the gut microbiota has been shown to influence not only chronic inflammation but also the regulation of incretin hormones and the function of insulin-producing pancreatic cells. Importantly, diabetic patients exhibit a lower abundance of butyrate-producing bacteria, short-chain fatty acids with anti-inflammatory and immune-modulating properties, which further exacerbates existing metabolic dysfunction [122]. The loss of these beneficial microbes represents a key link in the progressive deterioration of glycemic control.

The relationship between the microbiome and cancer is more complex and multifactorial. Current evidence suggests that certain bacterial communities may promote carcinogenesis through mechanisms such as chronic inflammation, production of genotoxic metabolites, and alteration of the tumor microenvironment. A paradigmatic example is colorectal cancer, where an overabundance of Fusobacterium nucleatum has been identified. This bacterium enhances local inflammation, disrupts cellular apoptosis, and stimulates tumor cell proliferation [123]. Additionally, the composition of the gut microbiome has been shown to significantly influence the efficacy of immuno-oncology therapies, such as immune checkpoint inhibitors. Recent studies reveal that specific microbial profiles are associated with improved responses to these treatments, opening the possibility of microbiome-targeted interventions to enhance clinical outcomes [124]. Moreover, in other cancers such as breast and liver cancer, the gut microbiota has been shown to modulate hormone metabolism and the detoxification of carcinogenic compounds, creating an environment conducive to malignant transformation [126].

Collectively, these findings highlight the relevance of the microbiome as a systemic modulator of metabolic and immune health. Therefore, studying the microbiome not only deepens our understanding of chronic diseases but also offers a promising platform for the development of personalized interventions, such as therapeutic diets, probiotics, prebiotics, or even fecal microbiota transplantation, that could significantly improve the prevention and treatment of these conditions.

## 3. Therapeutic Perspectives

### 3.1. Microbiome Profiles and Personalized Medicine

Microbial profiling represents a fundamental tool in precision medicine, enabling the understanding of how microbiome composition and function influence a patient’s response to various treatments. This perspective is particularly valuable in immunological diseases, certain types of cancer, and metabolic disorders [82].

In oncology, for instance, it has been observed that the response to immunotherapies, such as immune checkpoint inhibitors, varies significantly depending on the gut microbiome. Species such as *Akkermansia muciniphila* and *Faecalibacterium prausnitzii* have been associated with improved therapeutic outcomes, suggesting that microbiome modulation before treatment may enhance clinical responses [169]. This evidence has driven the development of intervention strategies based on the patient’s microbial profile, ranging from dietary modifications to personalized symbiotic and fecal microbiota transplantation (FMT). Collectively, these approaches constitute truly personalized medicine, in which interventions are tailored to each specific intestinal ecosystem [170].

### 3.2. Current Challenges and Future Research Directions

The interaction between the human microbiome and the immune system has emerged as one of the most transformative discoveries in 21st-century biology, profoundly transforming our understanding of human physiology and the origin of numerous diseases, with evidence showing that the gut microbiota performs fundamental roles in immune regulation, maintenance of epithelial integrity, and systemic homeostasis, while dysbiosis can trigger clinically relevant consequences in infectious, chronic, autoimmune, oncological, and neurological diseases [41,158]. Over the past decade, research has revealed the microbiome as a key regulator of immune cell maturation, cytokine secretion, and epithelial barrier function [140] and has driven the development of interventions including probiotics, prebiotics, symbiotics, and fecal microbiota transplantation (FMT), which have shown promising results in restoring microbial balance and regulating immune responses [141,167,168]. Microbiome-modulated immunotherapy, particularly in oncology, demonstrates that specific gut microorganisms influence the efficacy of immune checkpoint inhibitors, and landmark trials have shown that FMT from immunotherapy responders can confer benefits to previously non-responsive individuals [142,168]. In recurrent *Clostridioides difficile* infection, FMT has emerged as an effective strategy to restore eubiosis, and it is being explored in autoimmune diseases, cancer, and metabolic disorders, demonstrating potential in modulating immune responses, enhancing gut barrier function, and improving clinical outcomes [167,168]. Gene-editing technologies, such as CRISPR-Cas, allow precise interventions in microbiome composition and function, eliminating antibiotic resistance genes such as *blaNDM-1* or *mcr-1* without altering overall microbial structure and modulating microbial metabolic genes for therapeutic purposes [63,160,161], with recent advances highlighting the transformative potential of CRISPR-based interventions in microbiome engineering, including highly efficient base editing in gut *Bacteroides* spp., enabling targeted nucleotide conversions, multiplex editing across carbohydrate metabolism genes, and extending applicability to several commensal species [124,126]. Innovative delivery platforms, including phage-based systems, have enabled strain-specific depletion and targeted genomic deletions in complex communities, such as engineered bacteriophage M13 delivering CRISPR-Cas9 into *Escherichia coli* within the murine gastrointestinal tract, and CRISPR-Cas12a delivered via temperate λ phages discriminating microbial cells at single-nucleotide resolution, allowing highly specific elimination of bacterial strains [172,173,174]. Microbial metabolites, especially short-chain fatty acids (SCFAs), modulate the immune system by promoting regulatory T cell differentiation, reducing inflammatory cytokines such as IL-6 and TNF-α, and strengthening the epithelial barrier via proteins like claudins and ZO-1, with alterations linked to inflammatory, autoimmune, metabolic, and neurodegenerative diseases [162,163,164]. Profiling the microbiome for diagnostic and prognostic purposes, including microbial signatures associated with Crohn’s disease, multiple sclerosis, type 2 diabetes, and colorectal cancer, enables early intervention and personalized therapeutic strategies, particularly in oncology and immunotherapy [159,165,166], while early microbial colonization influenced by delivery mode, breastfeeding, and antibiotic exposure significantly shapes immune development [84,133]. The microbiome also modulates neuroimmune functions via the gut–brain axis, where metabolites such as propionate and tryptophan derivatives affect neuroinflammation, behavior, and neuronal plasticity, opening frontiers for psychobiotics and microbiota-targeted dietary interventions [108,135]. Despite these advances, clinical application of microbiome-based strategies faces significant challenges, including high interindividual variability, limited predictive capacity, incomplete understanding of host-microbiome mechanisms, regulatory and ethical constraints, and difficulties in standardizing interventions such as FMT and symbiotics [152,153]. Addressing these challenges will require advanced technologies for precise, dynamic, and context-specific microbiome characterization, incorporating artificial intelligence and machine learning for analyzing large-scale clinical and multi-omics data [156], as well as robust predictive models integrating genomic, metagenomic, transcriptomic, and metabolomic information to optimize therapeutic interventions, maximize efficacy, and minimize adverse effects. Ethical and social considerations, including equitable access and responsible management of genomic and microbiological data, alongside continuous medical education and public awareness initiatives, will be critical for the responsible and sustainable integration of microbiome-based therapies into healthcare systems [152,153].

## 4. Conclusions

The human microbiome has been established as an essential regulator of immunity, epithelial integrity, and systemic homeostasis. Its balance is fundamental to host health, while its dysbiosis has been linked to inflammatory, autoimmune, metabolic, and neurodegenerative diseases. This review reaffirms the microbiome as a functional organ, capable of modulating immune responses, generating bioactive metabolites, emitting molecular signals, and maintaining the integrity of the intestinal mucosal barrier. Recent advances in technologies, including CRISPR-Cas and omics platforms, have enabled more specific interventions, paving the way for precision microbiology with a therapeutic focus. Microbiome-based strategies, including symbiotic, FMT, probiotics, and microbial metabolites, represent promising avenues for personalized therapeutic approaches. The identification of microbial profiles as diagnostic and prognostic biomarkers further strengthens personalized strategies, allowing anticipation of disease progression and optimization of treatment responses, including immunotherapies and gut–brain axis interventions. Nevertheless, gaps remain in understanding interactions between the microbiota, host genetics, diet, and environment, as well as in standardization of clinical protocols and ethical and regulatory frameworks. Full integration of the microbiome into medicine requires overcoming these challenges through interdisciplinary, longitudinal, and personalized approaches, ultimately enabling predictive, preventive medicine grounded in the ecological and molecular complexity of the human being.

## Data Availability

No new data were created or analyzed in this study. Data sharing is not applicable.

## Abbreviations

TLR	Toll-like receptor
SCFA	Short-chain fatty acid
PRR	Pattern recognition receptor
AMP	Microbe-associated molecular pattern
